# Signal-Processing Procedure for Preliminary Concrete Anomaly Screening Based on Continuous Sliding Acoustic Excitation

**DOI:** 10.3390/s26196050

**Published:** 2026-09-24

**Authors:** Bochen Sheng, Shibin Teng, Qilong Xu, Wenlong Zhang

**Affiliations:** School of Smart City Engineering, Qingdao Huanghai University, Qingdao 266427, China

**Keywords:** concrete, anomaly screening, sliding acoustic excitation, signal processing, non-destructive testing

## Abstract

Internal discontinuities in concrete walls are hidden conditions that can compromise local structural capacity and durability. Rapid and objective on-site anomaly screening is therefore important for structural integrity evaluation. This paper presents a signal-processing procedure for preliminary concrete anomaly screening based on continuous sliding acoustic excitation. Acoustic signals were collected from local reference baseline regions and candidate anomaly regions along defined paths on an in-service reinforced-concrete flue wall. Time-domain waveforms, frequency-domain spectra, and short-time Fourier transform (STFT) distributions were combined to characterize the differences between reference baseline and candidate anomaly regions. Reference signals showed comparatively stable envelopes and concentrated spectral energy, whereas candidate anomaly regions exhibited greater amplitude dispersion, broader spectral distributions, and more pronounced non-stationary time–frequency features. A relative-variation screening workflow based on amplitude, spectral energy, and time–frequency features was formulated. The workflow supports the transition from subjective auditory judgment to quantitative signal analysis and is intended as an efficient Tier-1 preliminary screening approach for concrete structures. Because no destructive coring or paired NDT confirmation was performed on this in-service structure, the detected regions are designated strictly as candidate anomalies rather than confirmed voids.

## 1. Introduction

Concrete is widely used in buildings, transport infrastructure and municipal works because it combines high compressive strength, durability and construction flexibility [1,2]. During construction and service, inadequate consolidation, formwork leakage, entrapped air, moisture–temperature cycling and sustained loading can create cracks, voids or interfacial delamination [3]. These discontinuities reduce local continuity and may provide pathways for water, chlorides and carbon dioxide, thereby accelerating deterioration and reinforcement corrosion [4,5,6]. As existing infrastructure ages, rapid and low-cost non-destructive evaluation is increasingly important for structural health monitoring and quality assessment [7].

A void is a particularly difficult defect because it is hidden and its acoustic response depends on the surrounding concrete, cover depth and interface condition. Poor consolidation, retained bubbles and local leakage during casting are common causes [8,9]. A void can reduce local compactness, perturb stress transfer and increase the susceptibility of the surrounding material to moisture-related deterioration [10,11]. For concrete flue walls, reliable screening is therefore useful for locating areas that require confirmatory inspection, although an acoustic anomaly alone cannot determine void geometry or structural severity.

Available concrete NDT methods include ultrasonic testing [12,13], impact-echo [14,15], infrared thermography [16,17], ground-penetrating radar (GPR) [18] and impact- or sliding-acoustic inspection. Ultrasonic and impact-echo methods can provide information on internal interfaces and depth, but they require coupling or controlled impacts and their interpretation is affected by reinforcement and concrete heterogeneity [19,20]. Infrared thermography is rapid for shallow defects but depends strongly on heating and ambient conditions [21]. GPR covers large areas and can detect reinforcement and larger discontinuities, yet equipment cost and data interpretation are relatively demanding [22]. Acoustic inspection is portable and inexpensive, but conventional manual tapping remains operator-dependent. Recent studies have extracted time, frequency and time–frequency features from impact sounds and have explored machine-learning classification [23,24]. A practical gap remains: a transparent, low-computation procedure for continuous sliding acquisition that is suitable for rapid field screening and explicitly states its validation limits.

Sliding-acoustic inspection is attractive for preliminary field screening because the instrument is simple and a path can be surveyed continuously. However, the conventional auditory judgment of a clear or dull sound varies with the operator, contact force, surface roughness and background noise [25,26,27]. Relative signal features and reproducible preprocessing can reduce, but cannot eliminate, these sources of uncertainty.

Time–frequency tools such as wavelet packet decomposition and short-time Fourier transform (STFT) describe non-stationary acoustic responses [28,29], but their parameter choices and computational cost can hinder routine on-site use [30]. The present work therefore prioritizes an interpretable workflow based on three complementary features and a rule-based decision. The workflow is intended to identify candidate anomaly regions for follow-up testing, not to replace depth-resolved NDT methods.

This study evaluates sliding acoustic excitation on an existing reinforced-concrete flue wall, building on the authors’ previous studies of sliding-acoustic signal processing and acoustic autocorrelation analysis [31,32]. Time-domain amplitude, FFT spectral energy and STFT energy concentration are compared between local reference baseline regions and candidate anomaly regions.

Unlike laboratory studies with prefabricated defects, the field structure used here represents service conditions and construction heterogeneity. The study consequently emphasizes repeatability of acoustic patterns and transparent processing rather than exact estimation of defect dimensions.

A qualitative comparison with alternative NDT methods is also included. Sliding acoustic excitation offers low equipment cost, rapid path-wise coverage and minimal surface preparation, but it has shallower and less depth-specific sensitivity than ultrasonic testing, impact-echo or GPR. Its results remain sensitive to surface condition and operator motion. The appropriate engineering role is therefore preliminary screening followed by confirmatory NDT, not universal replacement of established methods.

The comparison in Table 1 summarizes the intended engineering role of each method. Sliding acoustic excitation has the lowest hardware burden and supports rapid path-wise screening, whereas ultrasonic testing and impact-echo provide more depth-related information. GPR is advantageous for larger areas and reinforcement mapping, while infrared thermography is most useful for shallow defects under suitable thermal contrast. The methods are complementary rather than interchangeable.

In light of these considerations, the primary contributions of this study are organized as follows:

(1) Continuous sliding-acoustic screening was evaluated on an in-service reinforced-concrete flue wall under realistic engineering surface and industrial background-noise conditions.
(2) Complementary time-domain, frequency-domain, and STFT representations were established to systematically characterize the acoustic divergence between local reference regions and candidate anomaly regions.
(3) Amplitude statistical distributions and dispersion measures were incorporated to improve the physical interpretability of contact acoustic variations.
(4) A multi-feature relative screening framework (R_A_, R_E_, and C_TF_) was formulated to serve strictly as an agile Tier-1 preliminary screening tool for prioritizing candidate suspect regions before confirmatory depth-resolved NDT diagnostics are deployed.

## 2. Test Methods

The experimental system consists of a handheld spherical hollow hammer, a portable recorder, and the existing reinforced-concrete flue wall (Figure 1). During each measurement, the hammer head is kept in close contact with the wall surface and continuously slid in a single direction, thereby generating continuous contact excitation rather than discrete single-point impacts. The inspection hammer used is equipped with a hardened steel ball head with a diameter of 20–25 mm, a total hammer mass of 0.15–0.25 kg, and features a telescopic handle. The recorder is placed at a predetermined distance from the sliding path.

The test subject is an in-service reinforced-concrete flue enclosure wall, with a wall thickness of 200 mm and a concrete strength grade of C30. Inside the wall, a double-layer orthogonal steel mesh is arranged using 10 mm diameter rebar with a spacing of 150 mm, and the concrete cover thickness is 30 mm.

The hammer was dragged once in one direction across each 1.5 m target path. The same operator performed all acquisitions and maintained the contact state and approximate motion as consistently as possible. The displayed records lasted approximately 5.5–7.0 s, corresponding to an average scanning velocity of approximately 0.21–0.27 m/s. Because each feature was calculated from the segmented samples and the amplitude was normalized to a local reference, duration changes primarily alter the time axis and the number of samples rather than the definition of RA, RE, or CTF. In the future, mechanical speed measuring devices or encoder-based speed recording systems will be used for subsequent verification.

Three independent recordings were collected for each of two representative paths (designated as groups I and II). Background noise was monitored continuously at the site. Each recording was segmented to isolate the effective sliding interval, and candidate short-duration spikes associated with loose surface debris were flagged during preprocessing. The primary dataset therefore comprises two survey paths and six recordings, while the supplementary Origin project contains additional field traces used for qualitative consistency checks. In accordance with operational preservation requirements, no independent coring or paired NDT confirmation was performed on this in-service structure. The complete specifications of the documented field dataset, acquisition parameters, and validation status are detailed in Table 2.

The raw WAV recordings (16 kHz, 16-bit mono PCM) were first preprocessed in Audacity (Version 3.4.2, Audacity Team) using Spectral Subtraction (Noise Reduction filter). An ambient noise profile was extracted from the initial 0.5 s static segment prior to sliding; filter parameters were set to Noise Reduction = 12 dB, Sensitivity = 6.0, and Frequency Smoothing = 3 frequency bands to suppress stationary industrial plant ventilation hum while preserving contact acoustic dynamics. The denoised traces were then imported into Origin for sliding window segmentation. To suppress high-frequency mechanical chattering caused by microscopic surface asperities, a symmetric 200-sample moving-average smoothing filter (corresponding to a 12.5 ms temporal window at 16 kHz) was applied to the rectified amplitude sequence.

FFT was used to describe the spectral distribution of selected waveform segments. The 16 kHz sampling rate gives a Nyquist frequency of 8 kHz. The FFT length was 256 points, with a rectangular window and amplitude correction. In the Origin (Version 2024, OriginLab Corporation, Northampton, MA, USA) project that retained the STFT settings, the STFT used a 256-sample Hann (Hanning) window with 128-sample overlap (50%), amplitude correction, and decibel output. At 16 kHz, these settings correspond to a 16 ms analysis window and an 8 ms step.

## 3. Results

### 3.1. Time Domain Analysis

To examine repeatability, two sliding paths along the flue wall were analyzed as groups I and II. Each path was recorded three times. The locations are treated as reference baseline and candidate anomaly regions based on repeated acoustic behavior and field observations, not as independently confirmed voids.

Across groups I and II, the waveform remained comparatively stable in the reference portions and exhibited larger, intermittent excursions in the candidate anomaly portions (Figure 2 and Figure 3). Short spikes labeled “sand and gravel” were treated as surface-interference events because they were brief and lacked persistence across adjacent windows. The transition between reference and candidate portions produced an energy change, but the magnitude varied between paths. These observations demonstrate repeatable acoustic contrast under the tested conditions, while their correspondence to a geometrically confirmed void remains unverified.

### 3.2. Amplitude Comparison and Analysis

Figure 4 and Figure 5 compare instantaneous amplitude distributions for the reference baseline and candidate anomaly portions. The candidate portions showed a wider range and larger dispersion than the reference baseline portions. This contrast is consistent with changes in local contact and wave scattering, but the data do not establish a unique resonance mechanism. Amplitude dispersion is therefore used as a screening feature rather than as a direct measure of void size or depth.

### 3.3. Frequency Domain Analysis

Figure 6 presents FFT spectra from the reference and candidate portions. The candidate traces contained stronger low-to-mid-frequency components, particularly within the 0–1500 Hz band used for R_E_, whereas the reference spectra were more concentrated. Peaks near approximately 400, 800 and 1200 Hz were observed in the displayed trace. These peaks are reported descriptively; without independent geometry, depth or modal measurements, they should not be attributed uniquely to void resonance.

The inset in Figure 6 enlarges the 0–1500 Hz interval to show the relative spectral contrast. The location and magnitude of individual peaks varied between recordings, which is expected for heterogeneous concrete and manually controlled contact.

The observed spectral enhancement is compatible with altered boundary conditions and scattering at a concrete–air interface, but other factors, including surface contact, reinforcement and wall geometry, may contribute. Spectral energy is therefore interpreted as a sensitive empirical indicator for screening under the tested configuration, not as a defect-specific physical constant.

### 3.4. Time Frequency Analysis

STFT was used to examine how acoustic energy evolved during sliding (Figure 7). The reference portions showed a comparatively continuous low-frequency distribution, whereas the candidate portions contained broader and more intermittent energy bands. The representation is useful for locating transient changes along a path. The horizontal axis denotes acquisition time and the vertical axis denotes frequency. Because speed was not mechanically controlled, the time coordinate should be interpreted as acquisition time rather than distance. The observed pattern is an acoustic anomaly indicator and does not by itself confirm a void.

### 3.5. Statistical Analysis of Amplitude Distribution

Figure 8 and Figure 9 show histograms of instantaneous voltage amplitude. The abscissa is voltage amplitude (mV), and the ordinate is the occurrence count within each amplitude bin, not acoustic frequency. The fitted normal curves are descriptive only. The reference distributions were narrower and more concentrated near zero, whereas the candidate distributions were wider. Means and standard deviations are reported to summarize dispersion, but no inferential test is claimed because the recordings are repeated traces from a small number of field paths.

The wider candidate distributions may result from a combination of internal discontinuities, surface contact variation and local debris. Accordingly, the amplitude distribution is used jointly with spectral and STFT features, and isolated spikes are removed during preprocessing.

The two groups showed the same qualitative ordering in distribution width, although their numerical ranges differed. This supports feature repeatability within the tested structure, but it is insufficient to estimate population-level sensitivity or specificity.

### 3.6. Analysis of Amplitude Dispersion Characteristics

Figure 10 summarizes instantaneous voltage amplitude using box plots. Boxes represent the 25th and 75th percentiles, the central line is the median, the marker is the mean, whiskers extend to 1.5 IQR, and points beyond the whiskers are outliers. Candidate anomaly portions had larger IQRs and more outliers than reference portions in both groups. These statistics quantify dispersion but do not independently verify the physical defect.

The consistent increase in dispersion indicates that the sliding signal is sensitive to changes in local structural or contact conditions. Since surface roughness and debris can produce similar isolated excursions, the box-plot result is interpreted as supporting evidence for screening, not as a standalone classification criterion.

The two groups therefore provide internal repeatability evidence for the amplitude features. External validation with known laboratory defects or a second NDT method is still required before the features can be assigned a universal diagnostic meaning.

### 3.7. Discriminative Analysis Based on Amplitude Features

The signal-processing procedure for preliminary concrete anomaly screening is illustrated in Figure 11 and comprises four sequential stages: (1) continuous sliding acoustic signal acquisition; (2) preprocessing, including sliding interval selection, trace-specific smoothing, and baseline normalization; (3) multi-domain feature extraction; and (4) multi-feature decision evaluation.

To reduce sensitivity to recording gain, microphone position, and batch-to-batch amplitude shifts, the procedure uses the following dimensionless relative-variation features:

(1) Amplitude Variation Rate (R_A_):

$$R_{A}=\frac{A_{v}-A_{n}}{A_{n}}\times 100\%$$

where A_v_ = (1/N) Σ|x_v_[i]| and A_n_ = (1/M) Σ|x_n_[j]| denote the mean absolute amplitude (MAA), equivalent to the rectified average signal value, of the target evaluation segment and the local reference baseline segment, respectively. No analytic Hilbert envelope was computed.

(2) Spectral Energy Variation Rate (R_E_):

$$R_{E}=\frac{E_{v}-E_{n}}{E_{n}}\times 100\%$$

where E_v_ and E_n_ denote the integrated FFT spectral energies within the selected low-frequency band of 0–1500 Hz for the target and reference baseline segments, respectively.

(4) Time–Frequency Energy Concentration Index (C_TF_):

$$C_{TF}=\frac{E_{max}}{E_{total}}=\frac{{max}_{k}\Sigma_{f}{\left|S(f,t_{k})\right|}^{2}}{\Sigma_{k}\Sigma_{f}{\left|S(f,t_{k})\right|}^{2}}$$

where S(f,t_k_) is the complex STFT coefficient at frequency bin f and time window t_k_. For each time window, the localized energy is obtained by summing |S(f,t_k_)|^2^ over all analyzed frequency bins. E_max_ is the largest of these window energies, and E_total is the sum of the window energies over the complete sliding segment.

Provisional Decision Logic:

For the current acquisition setup, the demonstration thresholds R_A_ ≥ 20%, R_E_ ≥ 15%, and C_TF_ ≥ 0.35 were established based on baseline noise-margin statistics. In the reference baseline segments across all repeated scans, normal surface roughness and background noise produced baseline fluctuations of R_A_ < 8.5%, R_E_ < 6.2%, and C_TF_ < 0.22. To establish an empirical safety margin of approximately 2.5–3 times the maximum baseline noise fluctuation while maintaining sensitivity to structural shifts, the demonstration thresholds were set accordingly. Validation status notice: these demonstration thresholds were established empirically for the specific contactor, 16 kHz recording setup, and C30 flue wall tested here. They have not been independently validated across diverse structural geometries, concrete compressive grades, or varying reinforcement configurations. A three-tier screening logic is implemented (Figure 11):

Reference/Acoustically Homogeneous Baseline Region: If zero or one criterion is satisfied, localized fluctuations are attributed to benign surface roughness or background noise, and the segment is retained within the reference-baseline category.

Suspected Anomaly: If two criteria are satisfied, the region is flagged as suspected, warranting cross-verification along adjacent scanning paths.

High-Probability Anomaly: If all three criteria are concurrently satisfied, the segment is prioritized for confirmatory inspection using independent depth-resolved NDT methods, such as ultrasonic pulse-echo or impact-echo testing.

These categorical outputs are acoustic anomaly-screening indicators rather than proof of void presence, geometry, or structural severity. The thresholds require recalibration when the acquisition hardware, wall geometry, surface condition, or ambient acoustic environment changes. If a stable reference-baseline segment cannot be identified, quantitative classification should be suspended in favor of qualitative spatial-contrast mapping.

## 4. Discussion

The experimental results in Figure 2, Figure 3, Figure 4, Figure 5, Figure 6, Figure 7, Figure 8, Figure 9, Figure 10 and Figure 11 demonstrate consistent acoustic contrast between reference regions and candidate anomaly regions under continuous sliding excitation. In the absence of destructive core samples on this in-service structure, the physical validity of the detected acoustic anomalies was evaluated by comparing specific numerical signatures with the established elastodynamic delamination literature. In concrete plate elastodynamics, shallow discontinuities decouple the near-surface concrete layer, creating a localized flexural resonator whose fundamental vibration frequency drops into the 300–1400 Hz range, distinctly separated from solid wall thickness modes (>10 kHz). The low-frequency spectral amplification and localized peaks observed near 400, 800, and 1200 Hz (Figure 6) closely match the flexural resonance bands reported by Yan et al. [14] (350–900 Hz for concrete void percussion) and Gan et al. [3] (400–1300 Hz for subsurface void acoustic–structure interaction). Furthermore, the massive acoustic impedance mismatch between concrete (Z approximately 8.0 × 10^6^ kg/(m^2^·s)) and air (Z approximately 415 kg/(m^2^·s)) reflects over 99.9% of acoustic wave energy back into the surface layer, physically generating sustained, high-energy STFT bursts (C_TF_ ≥ 0.35) lasting across multiple adjacent time windows.

Potential non-structural confounding factors were systematically evaluated. The flue wall contains an orthogonal double-layer Φ10 mm rebar mesh with 30 mm cover and 150 mm spacing. Fully bonded rebar stiffens the concrete matrix locally and produces acoustic wave scattering primarily at high frequencies (>10–20 kHz), rather than localized flexural resonance bursts in the 0–1500 Hz band. Loose surface sand particles produce transient contact clicks (<20 ms) with broadband profiles (>4000 Hz); these are reduced by the 200-sample moving-average filter and the multi-window STFT persistence criterion. The indoor flue wall was sheltered from direct weather with stable ambient humidity. Moisture gradients generally induce broad, diffuse attenuation across structural sections rather than sharp, localized anomalies along a continuous scanning line. These considerations support, but do not prove, the candidate-anomaly interpretation.

Quantitative classification metrics, such as confusion matrices or ROC curves, are deliberately omitted. On this in-service wall without core-drilling ground truth, adjacent sliding windows along the same scan path are spatially and kinematically autocorrelated. Calculating supervised performance metrics on correlated traces would constitute pseudo-replication and yield unverified, overly optimistic precision. Within-operator consistency is instead substantiated by the repeated, stable contrast profiles across independent passes shown in Figure 2, Figure 3 and Figure 12. Explicit limitations include the single C30 reinforced-concrete flue wall with 200 mm thickness, the lack of depth-resolved profiling, and manually maintained sliding velocity of approximately 0.21–0.27 m/s. The method is strictly intended as a rapid, low-cost Tier-1 preliminary anomaly screening tool. Suspect zones must be prioritized for Tier-2 confirmatory inspection using depth-resolved NDT methods before repair decisions are made.

## 5. Conclusions

This study presents a field-based signal-processing workflow for preliminary anomaly screening of concrete regions that exhibit acoustic contrast during continuous sliding excitation. Time-domain, FFT, and STFT representations were used to compare local reference baseline and candidate anomaly segments. The resulting multi-feature logic provides an interpretable basis for prioritizing follow-up inspection. Because independent defect confirmation and external validation were unavailable on this in-service structure, the method is designed strictly as a preliminary screening aid rather than a stand-alone diagnostic replacement for established NDT techniques. The main conclusions are as follows:

(1) Under the tested field conditions, candidate anomaly segments exhibited significantly larger amplitude dispersion, low-frequency spectral amplification (0–1500 Hz), and non-stationary time–frequency energy bursts compared to local reference baseline segments. These signatures demonstrate repeatable acoustic contrast but do not independently establish defect geometry, depth, or volumetric void severity.
(2) Joint consideration of the Mean Absolute Amplitude Variation Rate (R_A_), Spectral Energy Variation Rate (R_E_), and Time–Frequency Concentration Index (C_TF_) provides a dimensionless, objective screening framework. The demonstration thresholds (R_A_ ≥ 20%, R_E_ ≥ 15%, C_TF_ ≥ 0.35) are provisional values calibrated to the present acquisition hardware and baseline noise margins, and require recalibration for alternative structural conditions.
(3) Repeated sliding passes demonstrated qualitative consistency across traces within the investigated case. The primary limitations include the single 200 mm thick C30 reinforced-concrete wall, manually maintained scanning velocity (approximately 0.21–0.27 m/s), and lack of depth-resolved profiling. Independent ground truth, encoder-based velocity tracking, and multi-structural validation across different concrete grades and reinforcement densities are required before generalized diagnostic accuracy can be claimed. Consequently, this workflow serves strictly as an agile Tier-1 preliminary screening tool to guide subsequent confirmatory Tier-2 NDT testing.

## Figures and Tables

**Figure 1:**
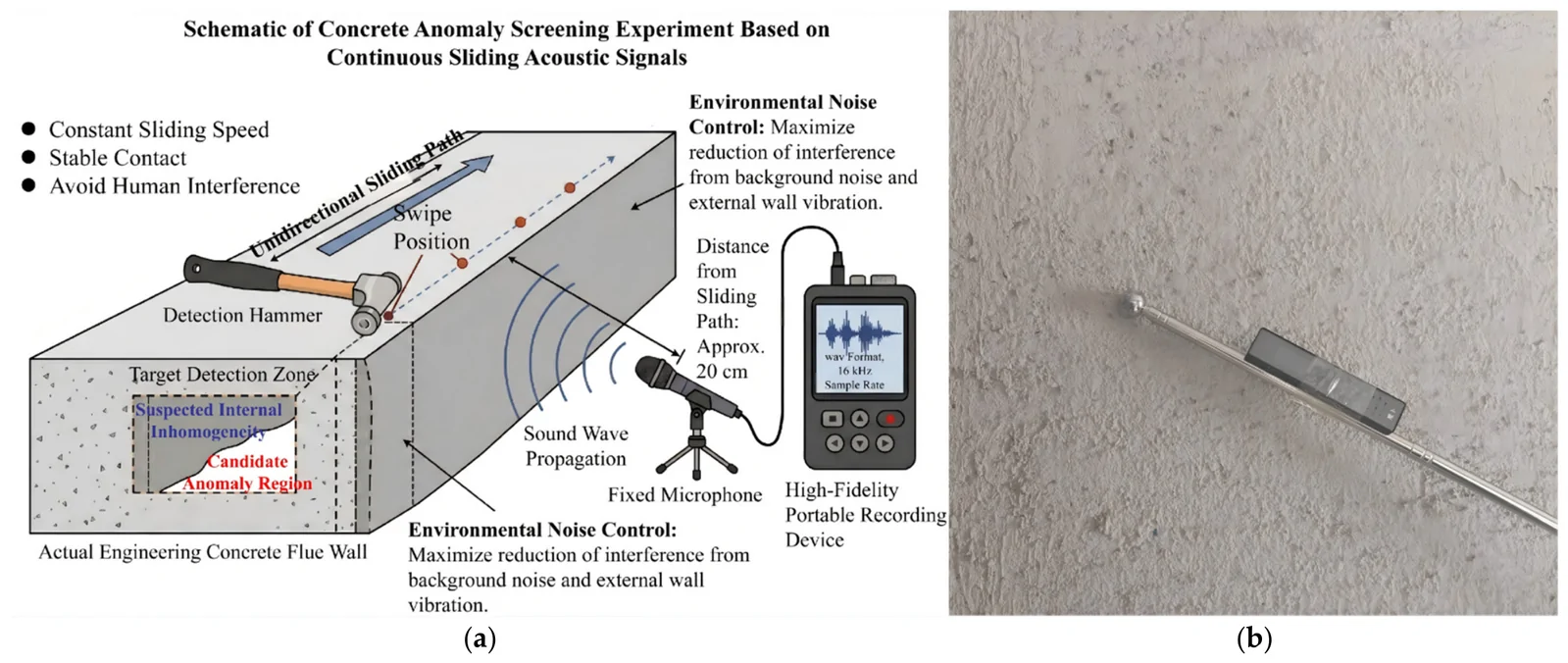
Experimental configuration and on-site testing: (**a**) schematic of continuous sliding acoustic excitation; (**b**) on-site test photograph.

**Figure 2:**
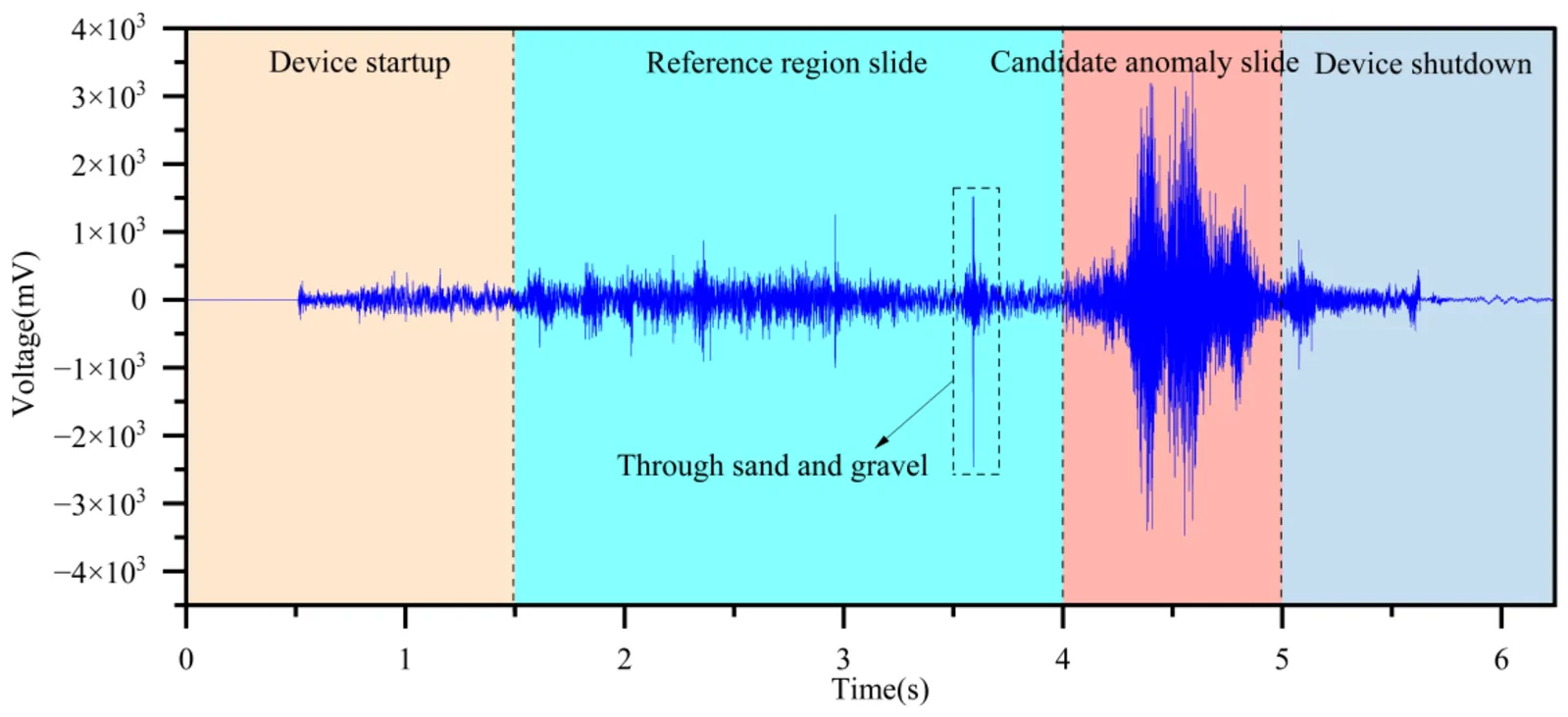
Time-domain waveform of acoustic signal during sliding from a reference region to a candidate anomaly region (group I).

**Figure 3:**
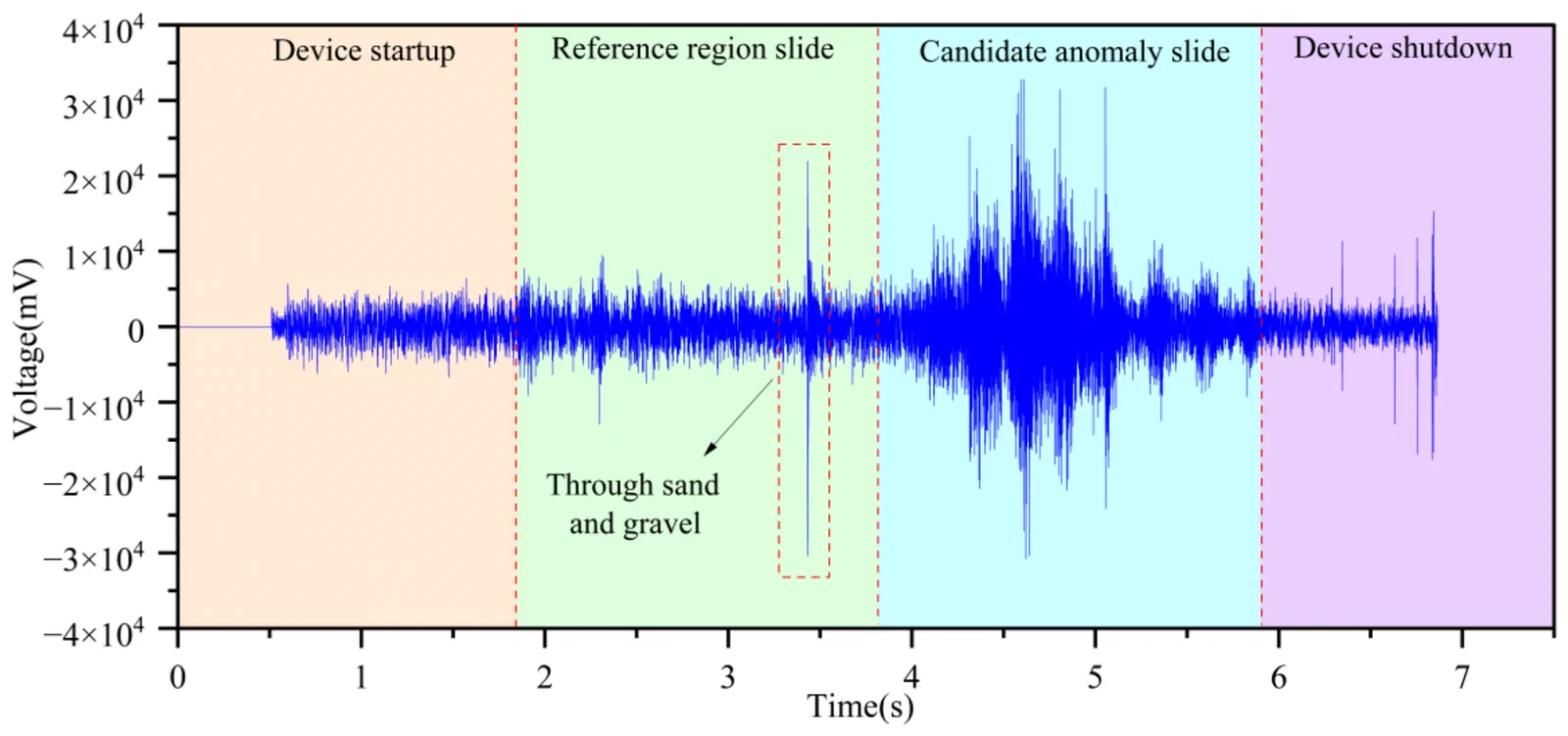
Time-domain waveform of acoustic signal during sliding from a reference region to a candidate anomaly region (group II).

**Figure 4:**
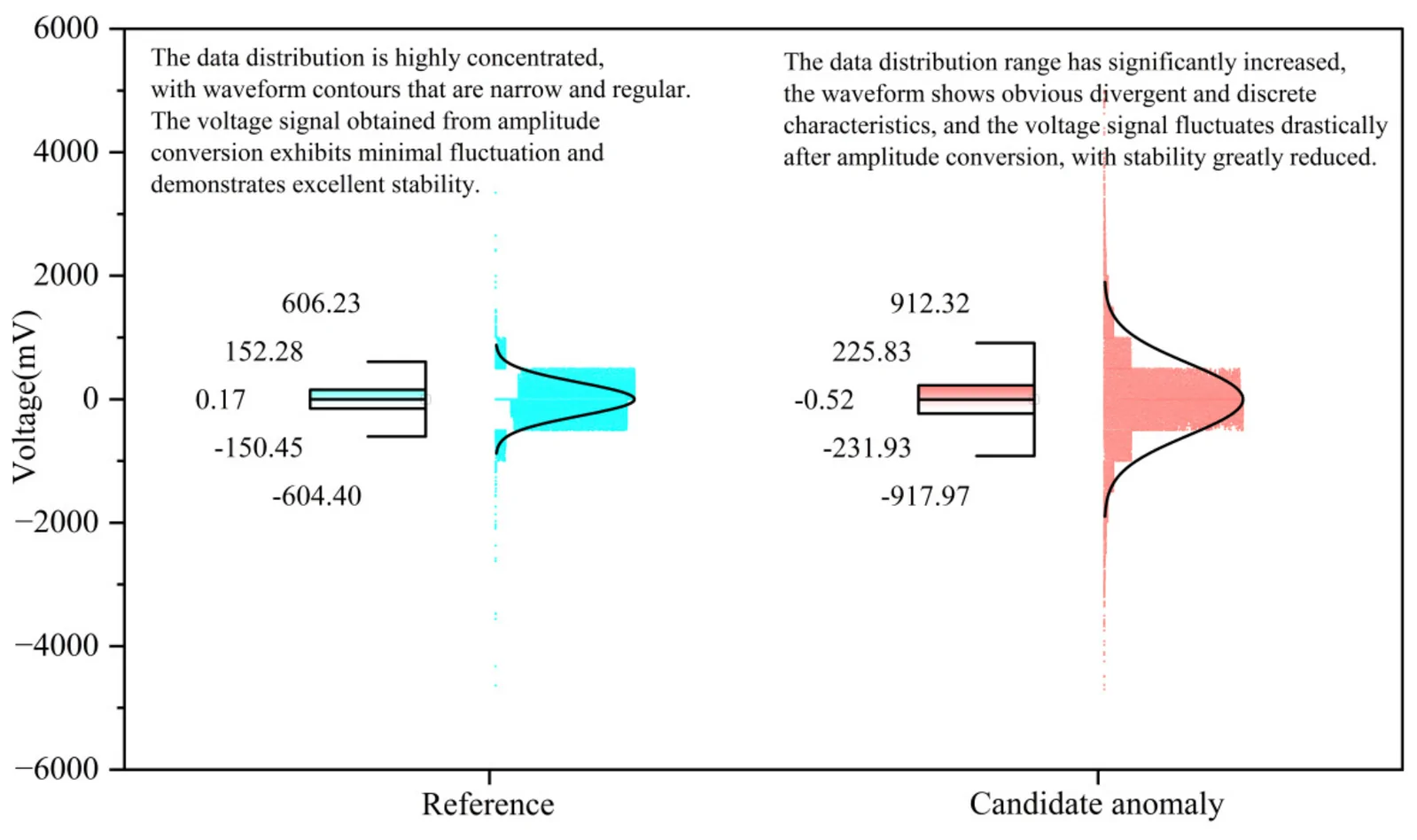
Instantaneous amplitude distributions for reference baseline and candidate anomaly regions (Group I).

**Figure 5:**
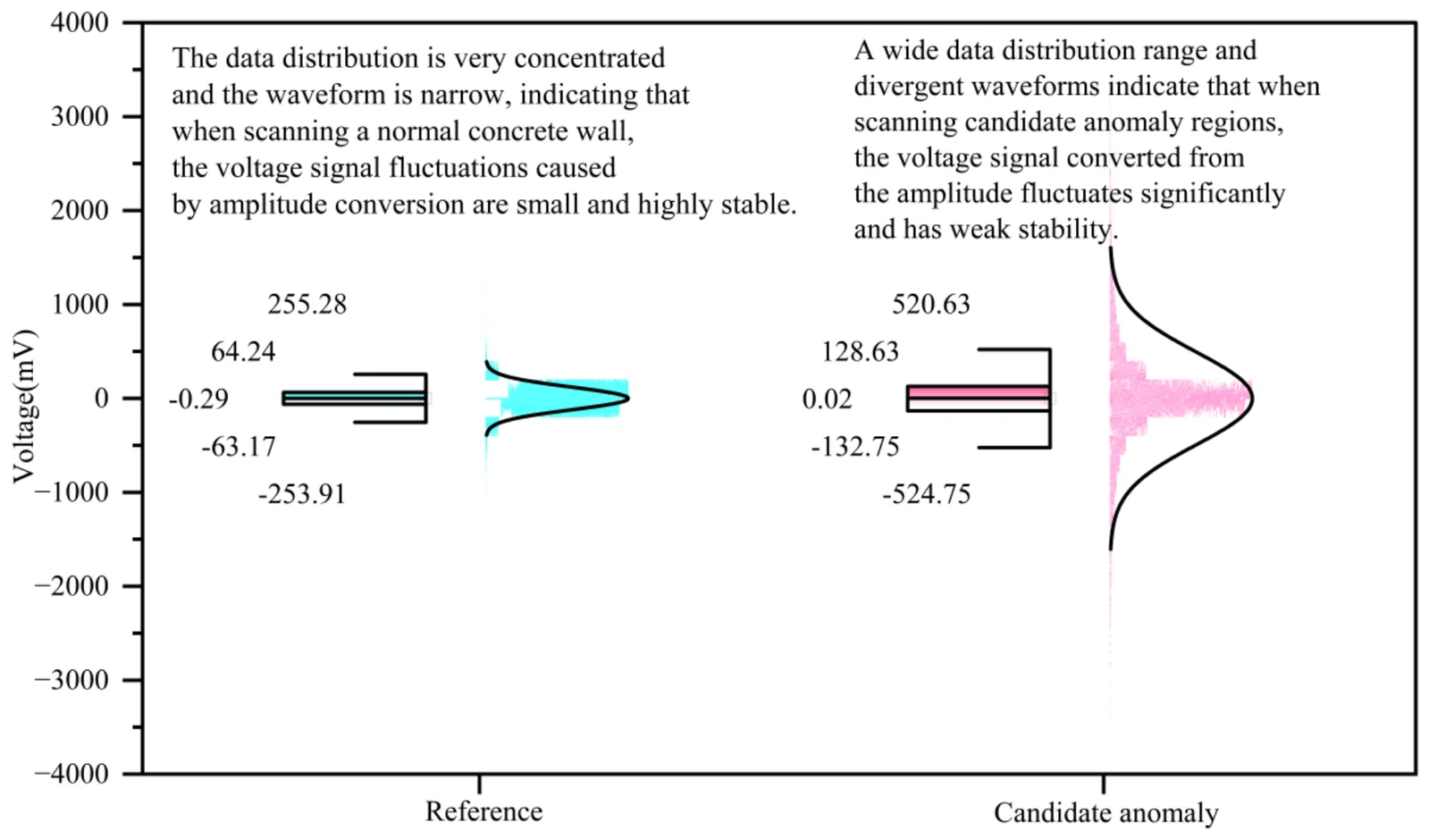
Instantaneous amplitude distributions for reference baseline and candidate anomaly regions (Group II).

**Figure 6:**
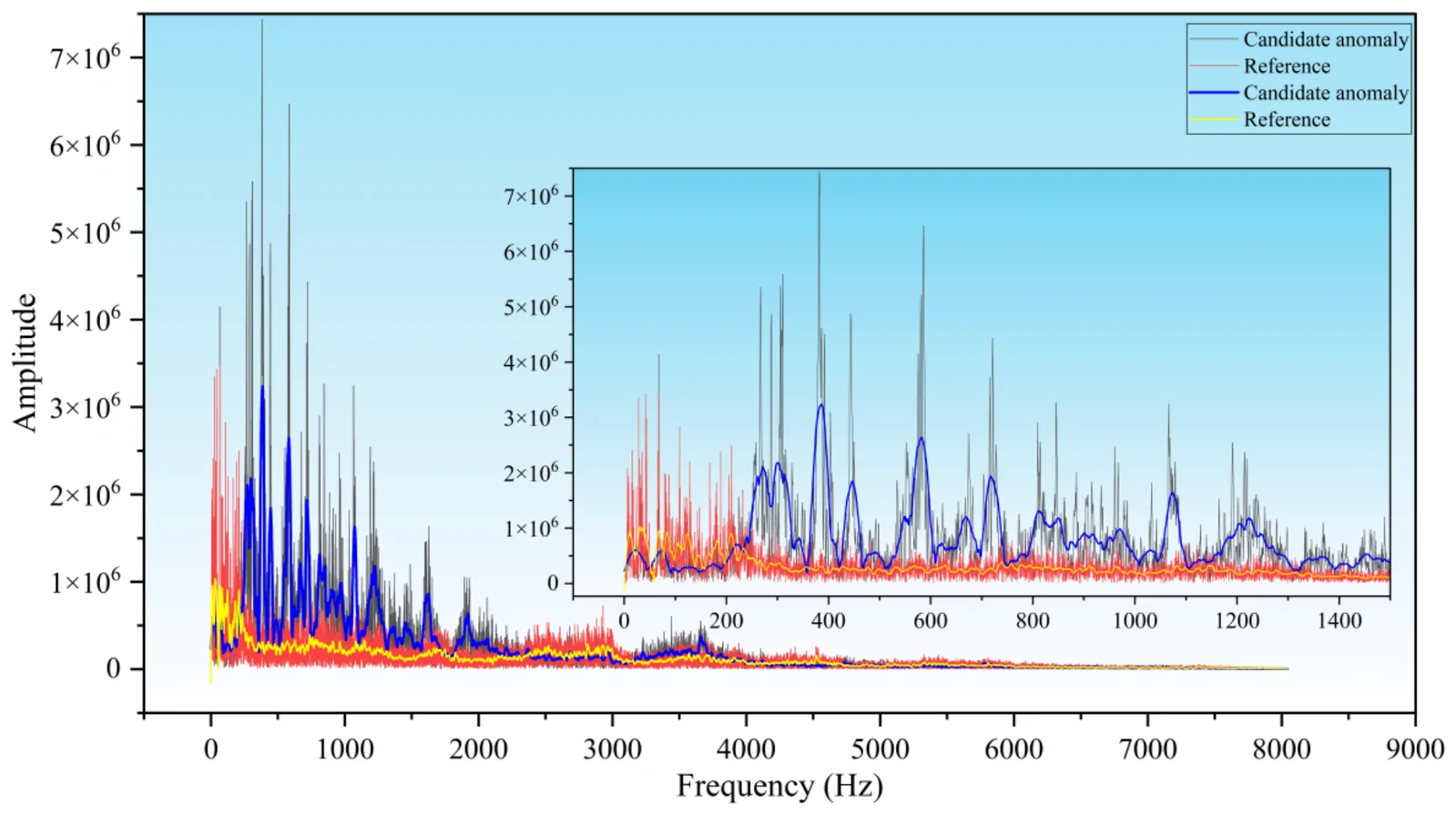
FFT spectra of reference and candidate anomaly acoustic signals in concrete.

**Figure 7:**
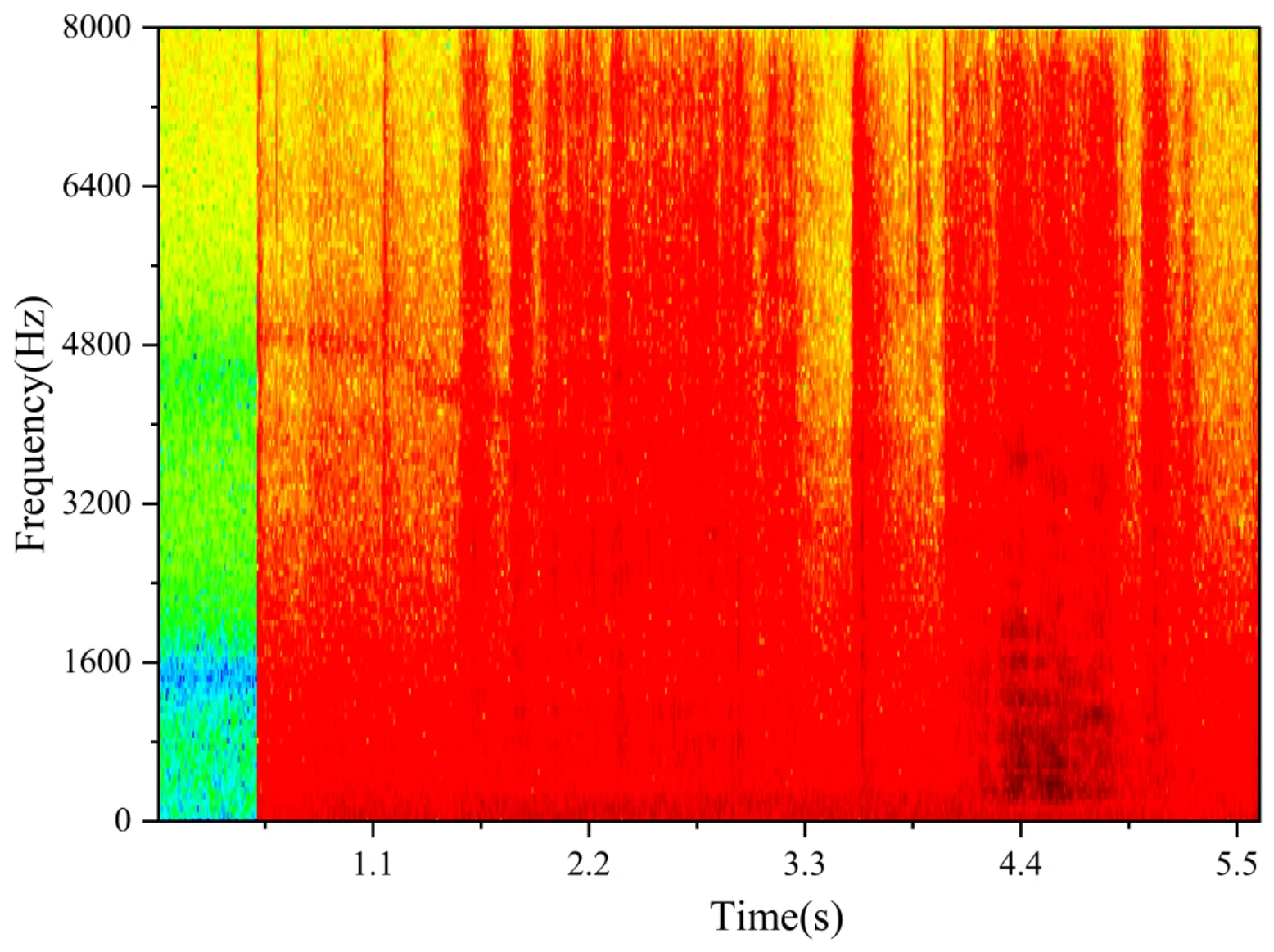
Time–frequency representation of the acoustic signal during continuous sliding.

**Figure 8:**
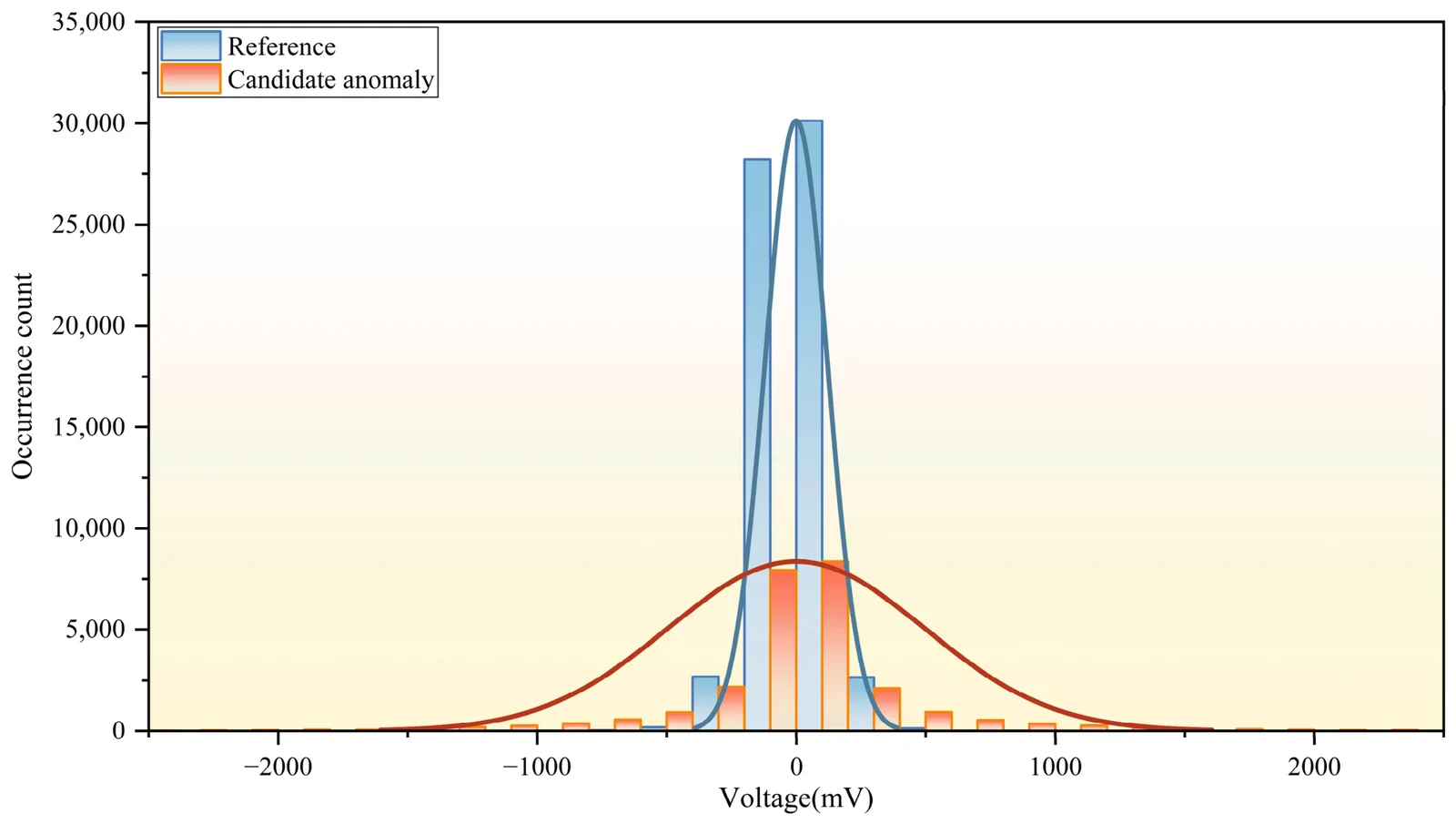
Distribution of instantaneous voltage amplitude in the first data group.

**Figure 9:**
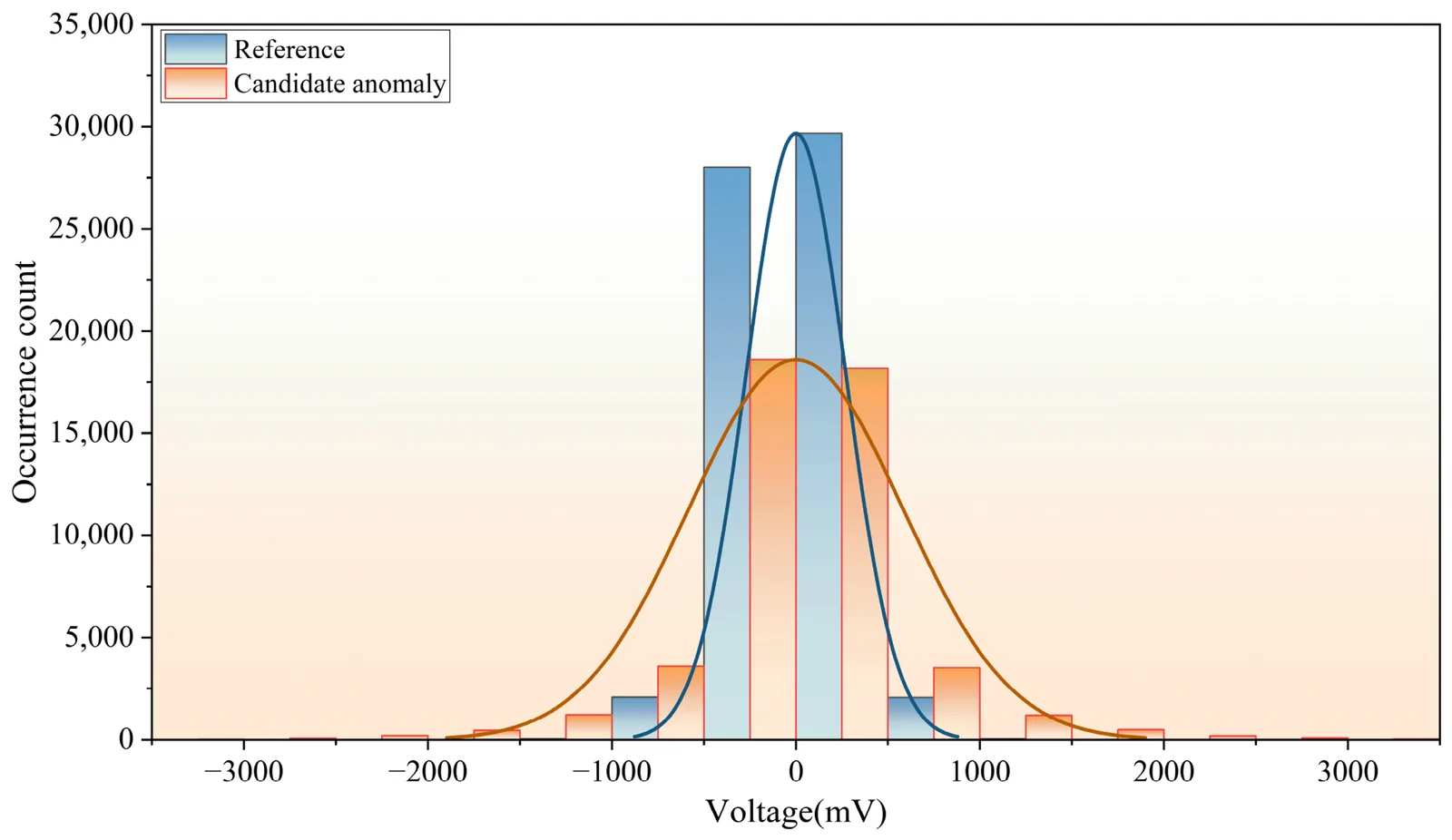
Distribution of instantaneous voltage amplitude in the second data group.

**Figure 10:**
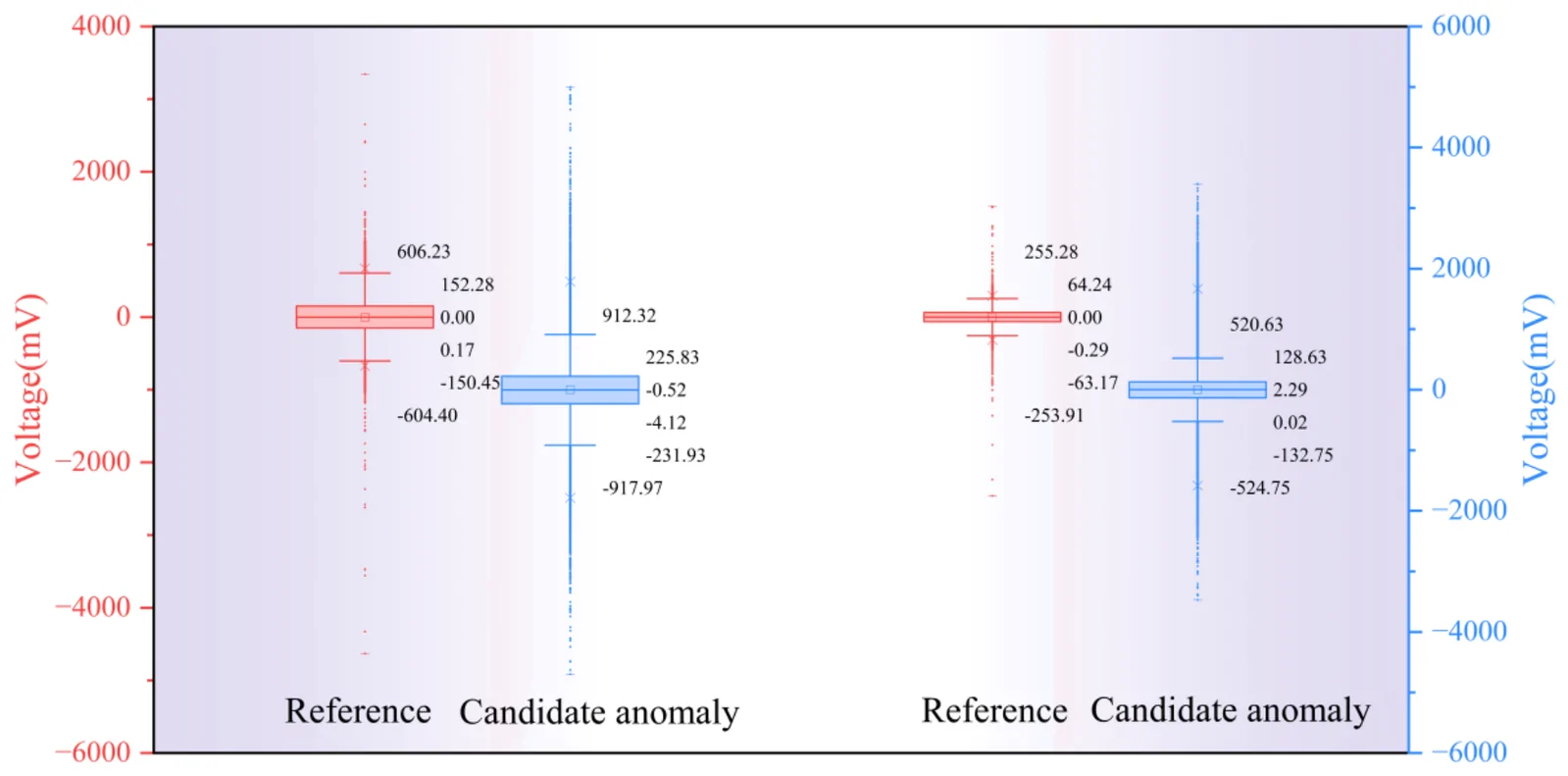
Box-plot comparison of instantaneous voltage amplitude in reference and candidate anomaly regions.

**Figure 11:**
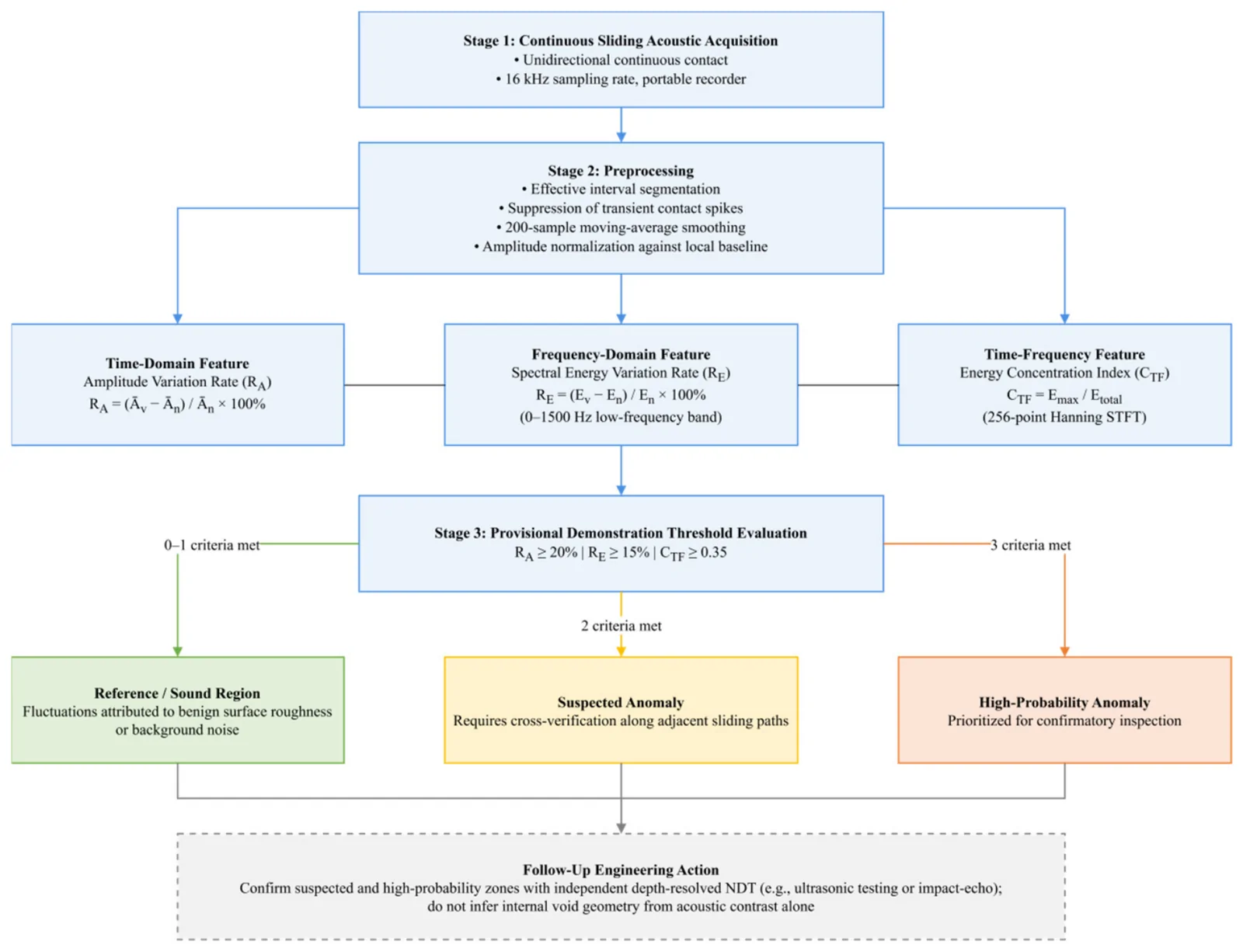
Multi-stage signal-processing and anomaly-screening workflow.

**Figure 12:**
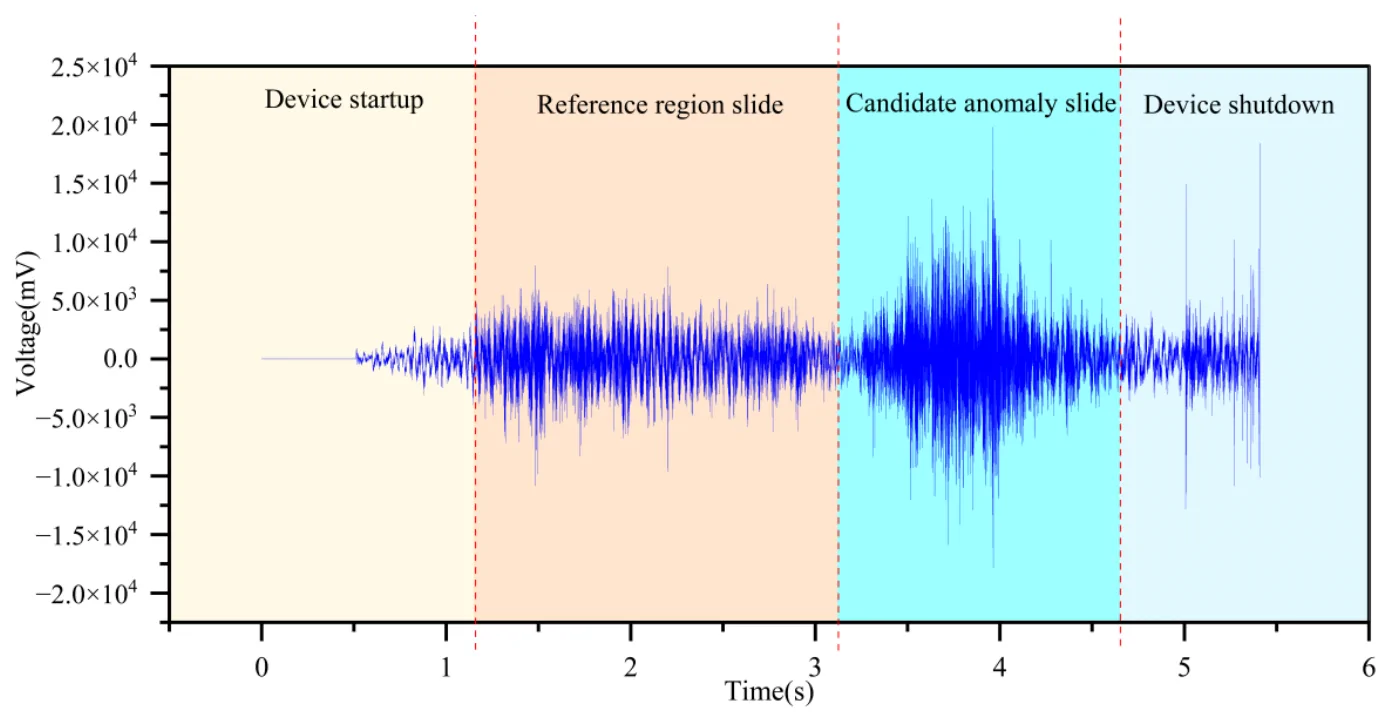
Supplementary time-domain acoustic waveforms along adjacent scanning paths.

**Table 1:** Qualitative comparison of the proposed sliding acoustic screening method with conventional NDT techniques.

Method	Cost/Hardware	Scanning Speed	Operator Dependence	Depth Sensitivity/Resolution	Primary Practical Role
Sliding acoustic excitation (this work)	Very low; portable recorder and spherical hammer	High; continuous path scanning	Moderate; uniform contact required	Surface and near-surface anomaly contrast; no depth-resolved imaging	Tier-1 preliminary screening
Ultrasonic pulse echo/array	High; transducers, couplant and acquisition unit	Low to moderate; grid or point inspection	Moderate; coupling and interpretation required	Depth-resolved internal interfaces; defect sizing subject to calibration	Tier-2 confirmation and sizing
Impact-echo	Moderate; impactor and receiver	Low; discrete point measurements	Moderate; impact condition-dependent	Thickness resonance and depth-related delamination assessment	Confirmatory thickness and delamination assessment
Ground-penetrating radar	High; antenna and processing unit	High; continuous cart scanning	Moderate; profile interpretation required	Rebar mapping and larger discontinuities; moisture-sensitive	Rebar mapping and macro-defect reconnaissance
Infrared thermography	Moderate to high; infrared camera and thermal excitation	Very high; full-field surface imaging	Low to moderate; thermal interpretation required	Very shallow thermal contrasts; weather and emissivity-dependent	Wide-area reconnaissance under suitable thermal gradients

**Table 2:** Summary of the documented field dataset and validation status.

Dataset/Processing Item	Documented Value	Validation/Interpretation Status
Field survey paths	2 representative paths (groups I and II)	Path count stated in the manuscript
Repeat recordings	3 recordings per path; 6 recordings in total	Repeated traces, not independent labeled specimens
Audio format	16 kHz, mono, 16-bit PCM; 1 raw WAV and 1 Audacity-denoised WAV pair supplied	Files available for processing verification
Independent defect labels	Not available	No coring or paired ultrasonic, impact-echo or GPR confirmation
Mechanical motion control	Not available; same operator performed all acquisitions	Speed, position and contact force were not encoder-recorded
FFT processing	256-point FFT; rectangular window; amplitude correction; 0–1500 Hz band used for R_E	Origin processing settings retained in the project file
STFT processing	256-sample Hanning window; 128-sample overlap (50%); dB amplitude output; 16 ms window and 8 ms step at 16 kHz	Origin processing settings retained in the project file
Audacity preprocessing	Spectral Subtraction (Noise Reduction filter); 0.5 s initial profile; 12 dB Reduction; Sensitivity 6.0; Frequency Smoothing 3 bands	Suppresses steady-state environmental plant hum while preserving transient contact acoustic dynamics
Origin smoothing	Symmetric 200-sample moving average (12.5 ms window at 16 kHz)	Applied to rectified absolute amplitudes to suppress high-frequency micro-chattering

## Data Availability

The data that support the findings of this study are available from the corresponding author upon reasonable request.
